# A Deep Learning Method for Vision Based Force Prediction of a Soft Fin Ray Gripper Using Simulation Data

**DOI:** 10.3389/frobt.2021.631371

**Published:** 2021-05-25

**Authors:** Daniel De Barrie, Manjari Pandya, Harit Pandya, Marc Hanheide, Khaled Elgeneidy

**Affiliations:** ^1^BioRobotics and Medical Technologies Laboratory, School of Engineering, University of Lincoln, Lincoln, United Kingdom; ^2^Lincoln Centre for Autonomous Systems Research (L-CAS), School of Computer Science, University of Lincoln, Lincoln, United Kingdom; ^3^A.D. Patel Institute of Technology, New Vallabh Vidyanagar, India; ^4^Compliant Robots and Devices (CoRD) Lab, School of Engineering, The Knowledge Hub Universities, Cairo, Egypt

**Keywords:** soft robotics, finite element analyses, fin ray, force prediction, deep learning CNN, soft robotic gripper, stress profiling, machine vision

## Abstract

Soft robotic grippers are increasingly desired in applications that involve grasping of complex and deformable objects. However, their flexible nature and non-linear dynamics makes the modelling and control difficult. Numerical techniques such as Finite Element Analysis (FEA) present an accurate way of modelling complex deformations. However, FEA approaches are computationally expensive and consequently challenging to employ for real-time control tasks. Existing analytical techniques simplify the modelling by approximating the deformed gripper geometry. Although this approach is less computationally demanding, it is limited in design scope and can lead to larger estimation errors. In this paper, we present a learning based framework that is able to predict contact forces as well as stress distribution from soft Fin Ray Effect (FRE) finger images in real-time. These images are used to learn internal representations for deformations using a deep neural encoder, which are further decoded to contact forces and stress maps using separate branches. The entire network is jointly learned in an end-to-end fashion. In order to address the challenge of having sufficient labelled data for training, we employ FEA to generate simulated images to supervise our framework. This leads to an accurate prediction, faster inference and availability of large and diverse data for better generalisability. Furthermore, our approach is able to predict a detailed stress distribution that can guide grasp planning, which would be particularly useful for delicate objects. Our proposed approach is validated by comparing the predicted contact forces to the computed ground-truth forces from FEA as well as real force sensor. We rigorously evaluate the performance of our approach under variations in contact point, object material, object shape, viewing angle, and level of occlusion.

## 1. Introduction

One of the primary applications of soft-robotics is adaptive grasping. The use of a passive, complaint structure which adapts to an object's geometry has showed great promise in applications when easily deformable and/or frangible objects are to be manoeuvred, such as in the automated harvesting of soft agri-food produce (Hemming et al., 2014). A great deal of work has been carried out within the broad field of soft-robotics, with many adaptive grippers having been developed (Rus and Tolley, 2015), including pneumatic (Hao et al., 2016), wire driven (Hassan et al., 2015), granular jamming (Brown et al., 2010), and compliant mechanism (Petković et al., 2013) based grippers. One such design which has shown great promise utilises what is commonly referred to as the “Fin Ray Effect” (FRE), originally presented by Festo Gmbh (Bannasch and Kniese, 2012; Festo, 2014).

**Figure 1:**
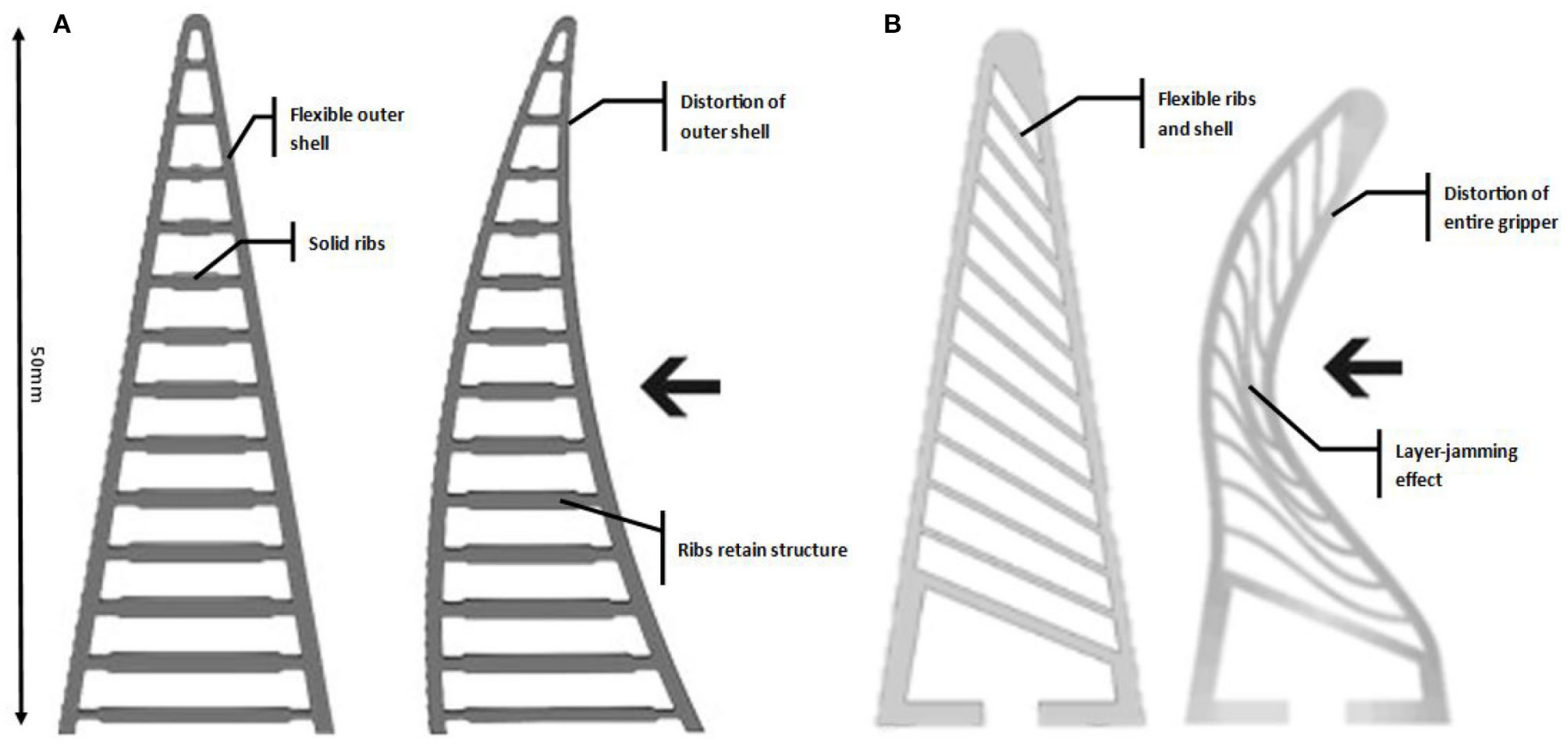
Fin-ray-effect gripper designs, **(A)** original fin ray gripper (Festo, 2020) presenting rigid ribs with a flexible outer layer, **(B)** modified fin ray gripper, the layer jamming effect can be seen (left) where the ribs “jam” together under deformation causing the effective stiffening of the gripper.

The FRE demonstrated by such grippers, mimics the biomechanical means in which fish fin operate. The result is a compliant grasping finger which requires no embedded actuation. The grippers deform when they come into contact with an object, adapting to the objects shape. The grippers are scalable and versatile in their application. FRE style grippers have been explored within literature for a range of applications. Our previous work (Elgeneidy et al., 2019) focused on the optimisation and characterisation of the FRE “cross-beams” to maximise the layer jamming effect, whilst maintaining high initial contact deformation. This work carried over into a Finite Element Analysis (FEA) of the 3D-printed FRE grippers, with our design being further optimised for layer jamming (Elgeneidy et al., 2020). The geometry of the gripper used in this work is based on our previous research (Elgeneidy et al., 2020). The gripper is completely flexible, directly printed in TPU flexible filament. The geometry differs from the original Festo design (Figure 1A) of the gripper though the use of flexible ribs which vary in angle, with a 3° ascending variance between them (Figure 1B). The result of these design changes is a gripper with variable stiffness thanks to the jamming of the ribs against one another under deformation. Analytical modelling approach for this fully soft FRE gripper is non trivial due to the layer jamming behavior causing a more non-linear response.

One of the drawbacks presiding over the field of soft-robotics is the use of sensors and contact modelling (Wang et al., 2018). The compliant structures do not lend themselves to traditional methods of modelling and control, as such new systems have needed to be developed. With the FRE grippers, a traditional actuator can be used; swapping out the rigid grippers for soft FRE grippers. The use of a rigid arm robotic system with soft-gripper end device, allows for the implementation of a traditional control system up to the end device, simplifying the procedure whilst maintain adaptive grasping. This approach, however, fails to consider the grippers own state (proprioception) and that of external stimuli (exteroception), such as the contact object, as the end device is still a passive compliant structure. Other numerical approaches, such as FEA modelling are often have larger processing time and which makes them difficult to adapt for real-time robotic applications, especially when modeling complex and deformable bodies without prior knowledge of the target objects.

**Figure 2:**
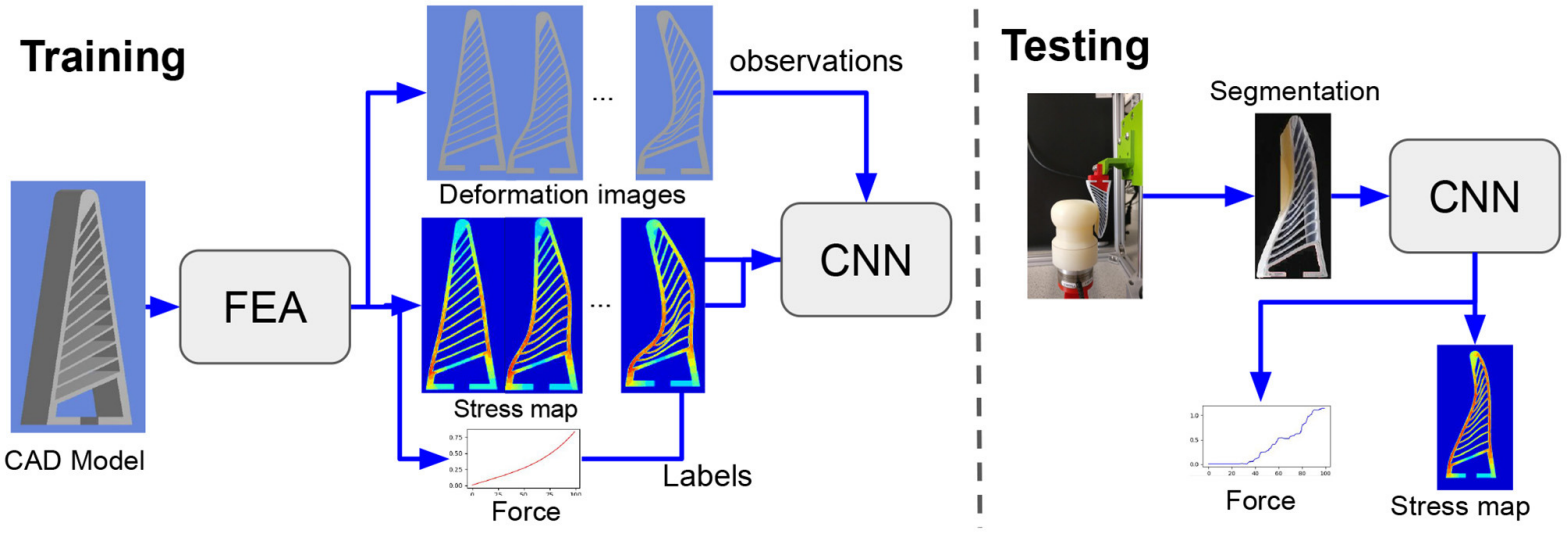
Pipeline: In this work we aim to learn the contact force and stress maps for a fin ray finger using visual input from an external camera. We employ images captured via the FEA simulation of the gripper to train our deep network. Once trained, the network could be employed to predict forces and stress in real time from the deformation images.

In this paper we present a machine learning based approach that is able to predict the contact forces for Fin Ray fingers in real-time using images from an external camera, circumventing the requirement of force sensors in a close loop control system. Figure 2 outlines the procedure used to conduct this work. Firstly a CAD rendering of the gripper is produced and simulated using the finite element method. The images and force measurements exported from this simulation is used to train the deep convolutional neural network (CNN). The verification of the system is performed by testing the gripper on a custom rig which records the resultant force caused by the displacement of the gripper onto the test object. A recording of the experiment is then segmented and fed into the CNN. The resultant is a stress map and the corresponding resultant forces. These results are then be verified against the experimental data and the FEA simulation.

## 2. Current Literature

One of the major difficulties presiding over the field of soft-robotics is accurate modelling of the gripper in real time. Complaint structures inherently do not lend themselves to traditional analytical techniques and the use of integrated sensors can compromise performance. FRE soft-grippers can be described as a 4D-printed structure, responding to external stimuli through layer jamming. Various techniques have been proposed developed in an attempt to model and characterise the behavior of such grippers (Zolfagharian et al., 2020). One such technique is to use a FEA approach. In terms of gripper optimisation, this is a well explored topic, with many successful examples of its application (Crooks et al., 2016; Basson et al., 2018; Emerson and Elgeneidy, 2020; Sun et al., 2020). Generally however, FEA is a computationally intensive process that is often time consuming, which makes its difficult as a means of providing real-time feedback control. A reduced order finite element model has been developed (Largilliere et al., 2015; Zhang et al., 2016). This work and more recent attempts (Katzschmann et al., 2019; Koehler et al., 2019) show promise, yet still presents several issues. Whilst reduced order FEA has been developed for soft robotics, Tonkens et al. (2020) it has yet to be experimentally verified and the technique has not yet been demonstrated on FRE grippers. The non-linear behaviour of this work's layer jamming fully flexible FRE gripper, would also provide an added degree of difficulty.

Work has been performed to produce a kinetostatic model of the FRE grippers for force estimation (Shan and Birglen, 2020). This approach constructs a pseudo rigid body mechanics model, with parameters taken from the geometry and a finite element model. This model's main purpose is aid in the task specific optimisation of the FRE gripper. A similar approach has been used in Kim et al. (2017) to develop a soft robotic glove and in the pneumatic gripper seen in Wang and Hirai (2017). All of these techniques, whilst helpful for optimisation, have not been utilised in the real-time control of the grippers, nor are they able to estimate the contact force without significant input data. The geometric complexity of the FRE gripper is limited here, with a uniform rigid cross beam structure. An analytical modelling approach would be significantly more complex for other FEA grippers utilising layer jamming behaviour.

Sensors have been incorporated into the design of many soft-grippers as a means of providing feedback for control. Some examples, where load cells have been incorporated into the design of compliant gripper (Abdeetedal and Kermani, 2018) or novel techniques such as the use of electro-conductive yarn (Matsuno et al., 2018), would not be suitable for the FRE finger style grippers. Other designs have moved away from the finger style approach, incorporating sensors in a larger compliant system (Petković et al., 2014). With regards to the finger style FRE grippers, sensors have placed on the surface for use within a haptic feedback system (Basson and Bright, 2019). These sensors however impact the way that gripper deforms on initial contact and demonstrated a slow system response. There has also been work conducted on an object recognition task based on deep convolutional neural networks (DCNNs) using a flexible sensor mounted to the surface of an FRE gripper (Gandarias et al., 2018). Whilst good results were achieved, the sensor placement has an impact on the compliant structure of the gripper and was shown to be less accurate when used with a fully flexible structure.

The challenge of connecting sensors to a soft-robotic gripper is nontrivial. Each sensor will require wires running to it, which must be channelled in such a way as to not impede the rest of the structure. To compensate for this, and the issues with reduced gripper deformability, the use of 3D-printing embedded soft-sensors (Shih et al., 2019) has been carried out, though this is still in its early stages. Soft electronic skins (Shih et al., 2020), containing a multitude of embedded sensors have also been examined for their application in the control and proprioception of soft-robots. Such sensors generally have a high resolution, requiring a complex algorithm to extract the useful information. Due to this, the use of machine learning techniques, in conjunction with soft skins, have shown promise. The complexity still proves problematic however, and soft skins have yet to make a great impact on soft-robotic control.

Tactile sensing is another means of control which has been shown to be effective, with some systems being able to estimate the deformation of a manipulated soft object (Sanchez et al., 2018). Such systems however primarily use a rigid structure, which can be modelled and controlled using traditional means, with only the contact pads providing the tactile feedback. The TacTip project (Ward-Cherrier et al., 2018) uses an optical approach to create 3D-printed tactile sensors. A series of pins, which mimic the structure of human skin, are created on a flexible surface. The vision system then determines a pressure map with a granularity based on the number and density of the pins. This is still limited however and would not be suited to FRE grippers.

Motivated from the ability of current learning based techniques to process higher dimensional data such as images, recent approaches such as Baghaei Naeini et al. (2020), She et al. (2020), Sferrazza et al. (2020), Han et al. (2018), and Liang et al. (2018) aim to employ deep learning based architectures to predict contact forces and stress maps. A few of the approaches rely on sensors (Han et al., 2018; She et al., 2020; Thuruthel et al., 2019) to predict the forces from sensory feedback, however, there is a limit to the amount of data which can be recorded, along with effecting the compliance of the gripper and the difficulty in wiring. For instance, a technique is purposed in She et al. (2020), whereby a computer vision based system incorporating a convolutional neural network is used to proprioception and exteroception of the wire driven soft-grippers. The system whilst effective, is still limited in the range of data captured and there no contact force estimation present. Buso et al. (2020) the measured colour changes in tandem with pressure readings were used to determine the force acting on a soft-robotic cushion. However, such sensors could not be used with FRE grippers due to the flexibility of the grippers. Alternative approach is to employ a vision based system such as a tactile sensor (Sferrazza et al., 2020) or a dynamic vision system (Baghaei Naeini et al., 2020) on gripper which observes the indentation of a deformable surface, rather than the gripper/compliant structure. The system utilises a machine learning system, trained via simulated data in order to estimate a contact force map. This system was shown to be effective but is limited to deformable objects and requires proximity for the vision system. The FRE design is naturally well-suited for vision-based systems, as unlike other grippers, there is no shell obscuring the internal structure. In this work, we employ an external camera to observe the deformations of the FRE fingers rather than objects to predict forces and stress map. Specifically, here we utilise our previously developed FEA model (Elgeneidy et al., 2020) to capture video simulation data of the deformation and colour coded stress-map to act as training data for a deep neural network. This is performed with aim of providing real-time contact stress mapping and resultant grasping force estimation of our layer jamming FRE gripper from visual data. Although in this work we focus only on the FRE gripper, our approach could be easily extended to other deforming gripper designs.

While the recent data driven techniques provide a promise to map forces from visual information, they require a large amount a data to do so. Thus, recent approaches employ simulators as surrogate for the real data (James et al., 2019; Zakharov et al., 2019). Liang et al. (2018) use FEA to predict stress maps in human organs, however they only showcase the results on simulation data. Similarly, for force estimation (Sferrazza et al., 2020) use simulation data from FEA to train their deep network. They present prediction results for real-world data, however only limit to cylindrical objects. In this paper we employ FEA to learn jointly learn contact forces and stress map from FRE deformation images. To the best of our knowledge, this is the first deep learning based approach that is able to predict contact forces as well stress profiles in real time from FRE deformation images. We also showcase the generalisation of our approach to arbitrarily shaped objects and to different materials. Finally we compare the predictions achieved by our approach vis-a-vis force sensor which showcases the efficacy of our approach.

## 3. FEA Procedure

The Finite Element Model utilised was previous developed for the optimisation of the Fin Ray rib structure, maximising the layer jamming effect for gripper force generation (Elgeneidy et al., 2020). This section will act as a summary of the model used, with the full details regarding the model and its validation being contained within the previous work. The model was created in the Static Structural package of ANSYS Workbench 19.3 (Ansys, Inc). This platform once configured allows for the gripper's deformation and resultant force generation to be determined and visually displayed from a range of input parameters. The outline of the procedure is that the gripper will remain static, with the object being displaced into the gripper; this simulates the effect of the gripper's deformation during an object grasp. The data and simulation video are captured during this and used to train the deep neural network. This section will break down the key defining aspects of the simulation namely the geometry, material definition, constraints, and result exportation.

### 3.1. Geometry

#### 3.1.1. Gripper

The geometry of the gripper was previously optimised within ANSYS Workbench. The gripper profile follows the basic FRE gripper outline, with the major adjustment from the original FESTO concept being the variable angled “ribs” designed for maximum layer jamming. The profile of the gripper is presented as a 2D CAD model, created in the ANSYS Workbench CAD package “Design Modeller.” The 2D profile is later extruded during simulation to give a 20 mm wide gripper.

#### 3.1.2. Objects

**Figure 3:**
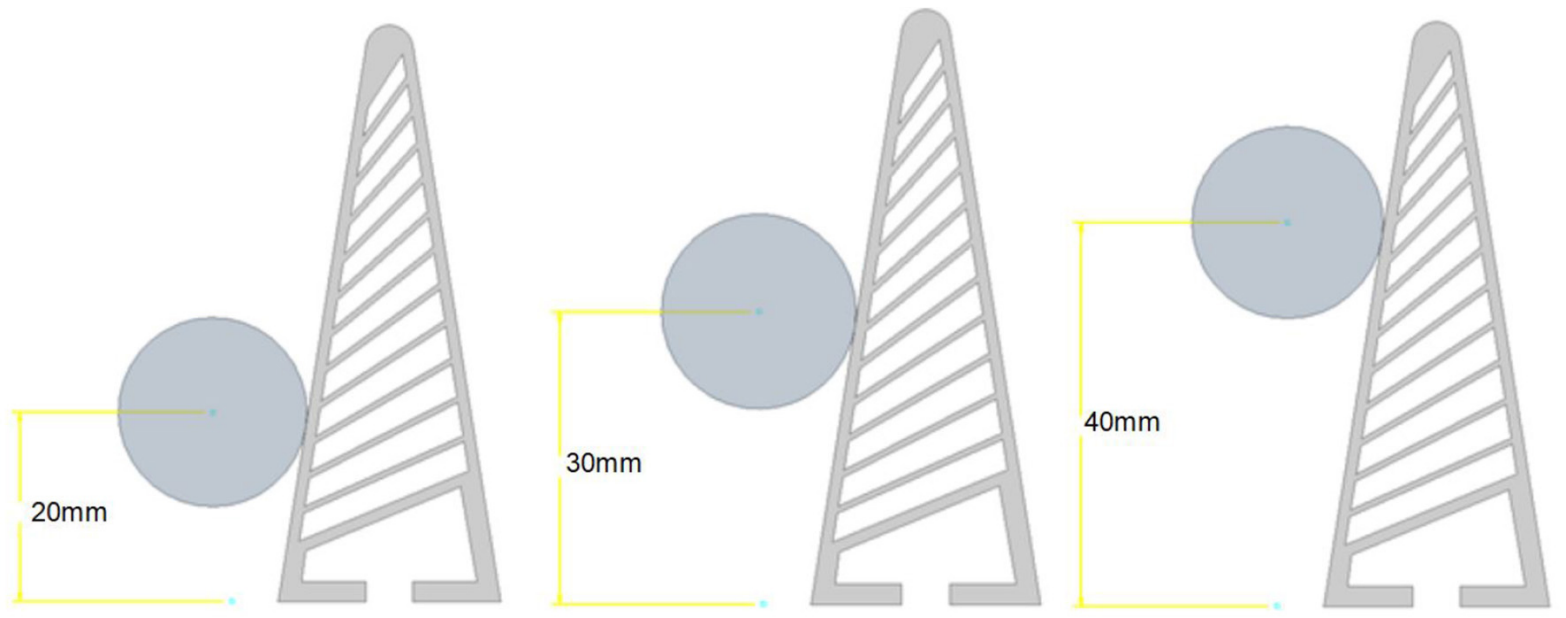
Object height increments. We capture the variations in point of contact by explicitly generating training simulations for object contacts at three different heights representing contact forces at low, mid, and high point.

The objects are defined in the same drawing as the gripper. Just as with the gripper, the 2D drawing is produced and later extruded to 20 mm during the simulation process. The objects contact the gripper at three height increments relative to the objects centre point and the base of the gripper (Figure 3). The height increments are 20, 30, and 40 mm.

**Figure 4:**
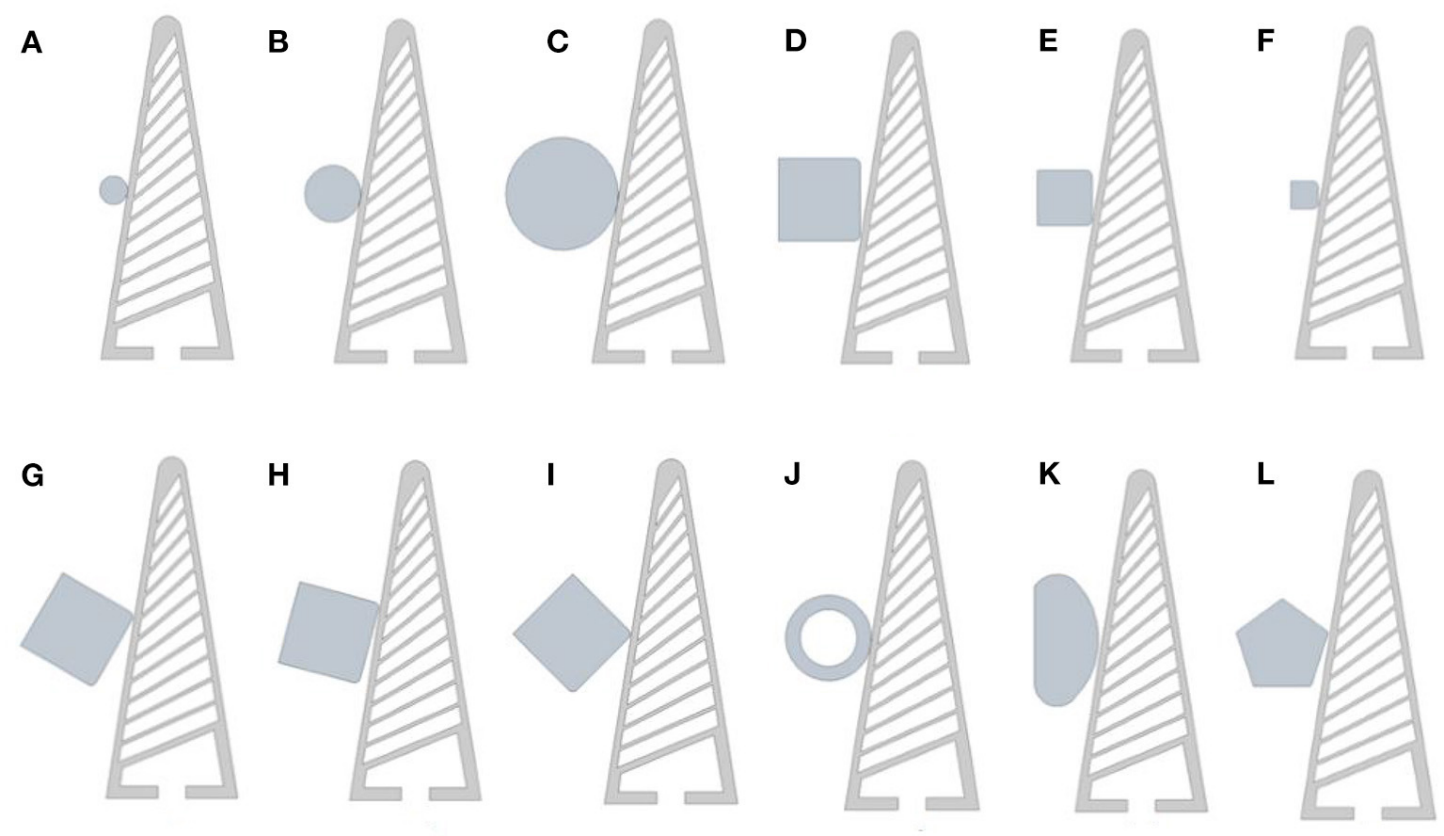
**(A)** 5 mm circle, **(B)** 10 mm circle, **(C)** 15 mm circle, **(D)** 15 mm square, **(E)** 10 mm square, **(F)** 5 mm square, **(G)** square offset 30°, **(H)** square offset 45°, **(I)** square offset 45°, **(J)** hollow ring (soft-object), **(K)** abstract test object, **(L)** pentagon test object.

The objects shown in Figure 4, comprise of some primary shapes with varying size and approach angles, along with more abstract shapes. Each of the shape is dimensioned around a centre point, used for the height increments, and with a constraint which ensures that the object's contact surface remains offset 0.1 mm to the angled contact face of the gripper. Three different size circles are used: 5 mm (Figure 4A), 10 mm (Figure 4B), 20 mm (Figure 4C). These are the simplest object as angle is indifferent and the contact area is distributed rather than concentrated around a small focal point. A small contact point can in some instances create excessive localised forces outside of the current material model limits. The square shapes for this reason feature a small fillet (0.5 mm) to counter this excessive localised stress. The squares are sized at 15 mm (Figure 4D), 10 mm (Figure 4E), and 5 mm (Figure 4F) and are angled at four 15° increments relative to the base of the gripper (Figures 4G–I). The material of the objects is by default “structural steel”^1^, meaning there is an inconsequential amount of deformation. To demonstrate the effect of varying the object hardness, a 15 mm hollow ring (Figure 4J) was used which allows for clear object deformation to be observed alongside the gripper distortion. This hollow object is also presented to the gripper at 3 height increments. The remaining objects (Figures 4K,L) are used to validate the effectiveness of the trained system. These objects were once again tested at the three height increments, with the object material defined as “structural steel.”

#### 3.1.3. Mesh Parameters

A uniform mesh is used on both the objects and the gripper. The mesh method uses the default “mechanical, programmed controlled,” with an element sizing of 0.5 mm. This level of granularity was found to be sufficient to run the simulation, whilst not being excessively time consuming. This was an important factor given the relatively large number of training and testing simulating videos which would be produced using this model. Further optimisation of the meshes could be conduced, but was not the primary focus of this work.

### 3.2. Material Definition

The FRE gripper is produced directly using fused deposition modelling (FDM) 3D-printing. The material used is a TPU based filament called NinjaFlex (Fenner Inc.)^2^, one of the most wildly used flexible filaments. NinjaFlex does not appear as a standard material within the ANSYS package, as such a custom model was produced using (Reppel and Weinberg, 2018) as a basis. The material model has been previously verified and is shown to closely approximate the behaviour of FDM printed NinjaFlex. For the soft object test, both structural steel and NinjaFlex were used alongside ANSYS material library's “neoprene rubber”^3^. The rubber material is significantly softer than the NinjaFlex gripper, resulting in much of the deformation occurring in the object rather than the gripper.

### 3.3. Contacts, Constraints, and Displacements

The simulation is set-up around defined conditions which aim to mimic the gripper interacting with objects. For the simulation to function correctly, any parts which may contact one another must be defined with a frictional relationship. This is the case for both the ribs of the FRE gripper and the contact surfaces of the object and gripper. This is defined with frictional coefficient of 0.15, modelled via Augmented Lagrange. The base of the gripper is locked under a static constraint. This allows for the gripper to flex within its elastic limits in *X* (horizontal direction normal to applied force), whilst the base remains static. With this constraint in place, it is the object which moves into the gripper, rather than the gripper to the object as would be the norm in the gripper's application. To this end the object is acted upon by a remote displacement of 10 mm in the *X* direction only, acting from the centre of the object. The other directional and rotational axis are fixed. In most cases this displacement is at the limit of what the simulation would allow. Exceeding this would oftentimes result in an excessive localised stresses, which cause the model to fail. It is assumed that this is due a combination of factors, including the use of a custom non-linear material model; however as this only occurs in over-stressed conditions, the issue has not been fully investigated.

### 3.4. Simulation

Using the model defined in this section, the simulation is run for each of the shape and material configuration. Videos of the gripper deformation, a log-scale stress map, and the resultant force data is exported for use in the CNN training detailed in the proceeding section.

## 4. Learning Force and Stress Profiles

### 4.1. Dataset

The objective of the proposed approach is to learn the contact forces and stress profile from visual observations to achieve real-time predictions for forces which are difficult to obtain via analytical and numerical methods. Using the FEA data collection procedure described in the previous section, we trained on 20,000 data samples obtained by varying size, height, and angle of contact for sphere and cube shapes. Each data sample comprises of a three channel deformation image (**O**), a stress map represented as a single channel segmentation image (**s**) and a contact force (*F*_*x*_) in the normal direction. In this paper, we focus on predicting the normal force, however our approach could easily be extended to prediction of other components of forces as well. As data prepossessing, the images were cropped, down-sampled and the fingers were segmented, before feeding to the CNN.

### 4.2. Network Architecture

**Figure 5:**
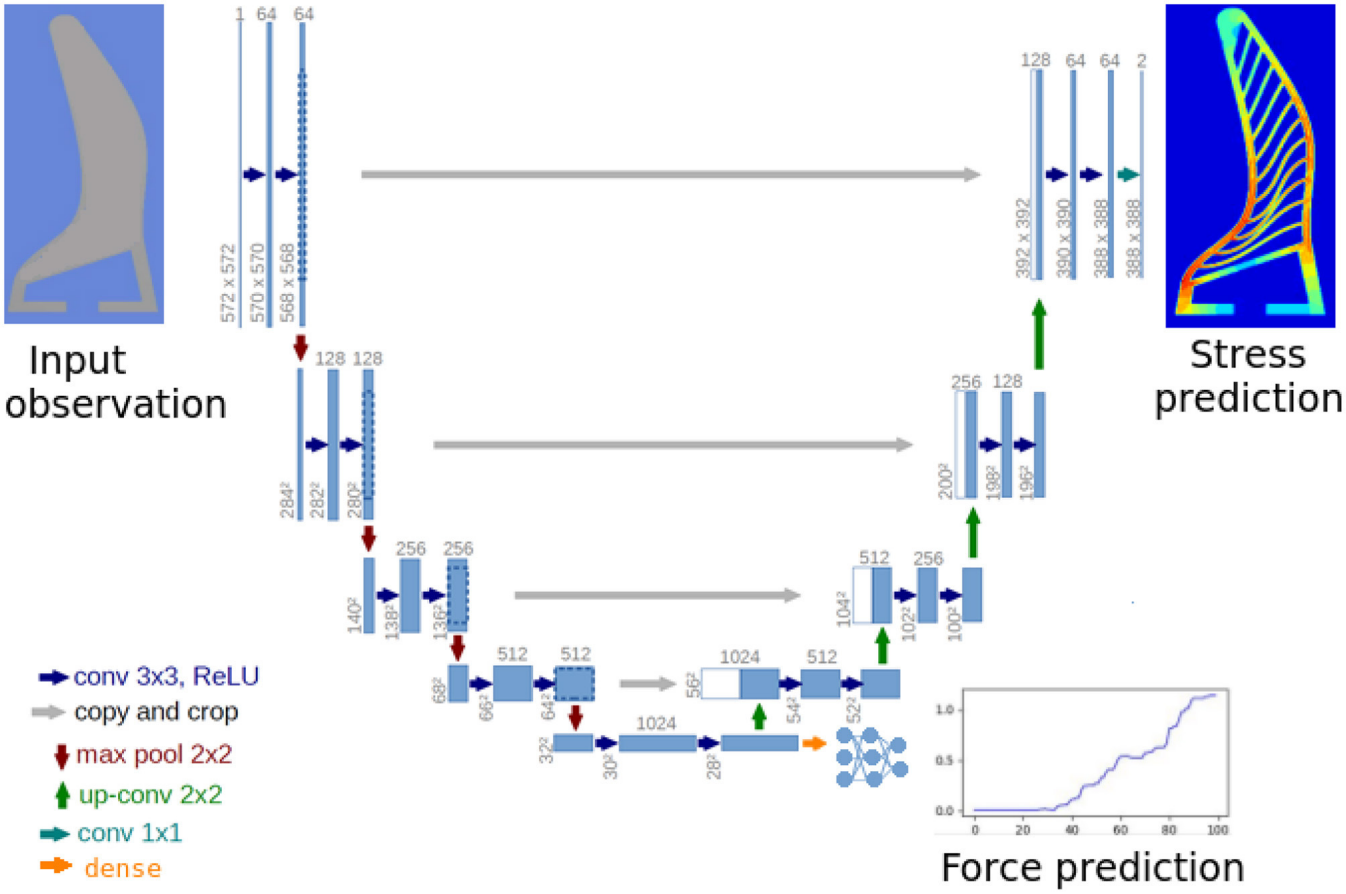
Network architecture: the encoder and decoder follow similar architecture as U-net (Ronneberger et al., 2015) including the skip connections between encoder and decoder. We add an additional branch for decoding forces from the intermediate representation. The force decoder consist of a dense fully connected neural network of depth five layers. The entire network is trained in an end-to-end fashion by optimising the joint loss function (Equation 1).

The network architecture is illustrated in Figure 5. It consists of three parts (i) Deformation encoder (**E**_θ_), (ii) Stress decoder (**D**_ϕ_) (iii) Force decoder (**D**_ψ_). Where, the deformation encoder takes in the deformation image and learns a latent representation (**h**) that could be decoded back to stress map (**S**) and forces (*F*_*x*_) using (**D**_ϕ_) and (**D**_ψ_). The encoder (**E**_θ_) follows the typical architecture of a convolutional network. It consists of the four stacks of two 3 × 3 convolution layers, each followed by a rectified linear unit (ReLU) and a 2 × 2 max pooling operation with stride 2 for downsampling. At each downsampling step we double the number of feature channels. The stress decoder is designed similar to the U-net expansion structure (Ronneberger et al., 2015) with skip connections. Every step in (**D**_ϕ_) consists of an upsampling of the feature map followed by a 2 × 2 convolution (“up-convolution”) that halves the number of feature channels and two 3 × 3 convolutions, each followed by a ReLU. Finally, force decoder is a dense feedforward network consisting of 5 fully connected layers of (128-64-32-8-2) neurons, respectively. The first fully connected layer has ssigmoid activation while others have ReLU activation. The latent features **h** are flattened before feeding to **D**_ψ_. At every step we decreased the number of parameters down to one in the last layer. There is a batch-normalisation layer placed after the initial fully-connected layers to refrain from overfitting.

### 4.3. Training Procedure

The entire network is trained in an end-to-end fashion, by minimising the following function over the training dataset, consisting of the individual mean squared loss over stress and force:

(1) $$\begin{array}{l} L=\underset{i}{\sum }\left(\alpha_{s}∥S_{i}-{\text{D}}_{\phi }\left({\text{E}}_{\theta }\left({\text{O}}_{i}\right)\right)∥+\alpha_{f}∥{F_{x}}_{i}-{\text{D}}_{\psi }\left({\text{E}}_{\theta }\left({\text{O}}_{i}\right)\right)∥\right)/N, \end{array}$$

The network is trained for 10 epochs on the training dataset, using Adam (Kingma and Ba, 2014) gradient decent approach with the learning rate of 10^−4^. The weights of individual loss functions α_*s*_ and α_*f*_ are selected as 1 and 10 to match the gradients from stress map and force predictions. The batch size *N* is selected as 16 according to GPU memory. We use a workstation with 64 GB RAM, Intel Xeon processor and Nvidia Geforce 2080 GPU to train and evaluate the system.

## 5. Experiments

For evaluating the proposed approach we perform a series of experiments focusing on generalisation of the network to different shapes and materials. We select the performance metrics as mean squared error for both the stress as well as force predictions.

### 5.1. Shape Generalisation

**Figure 6:**
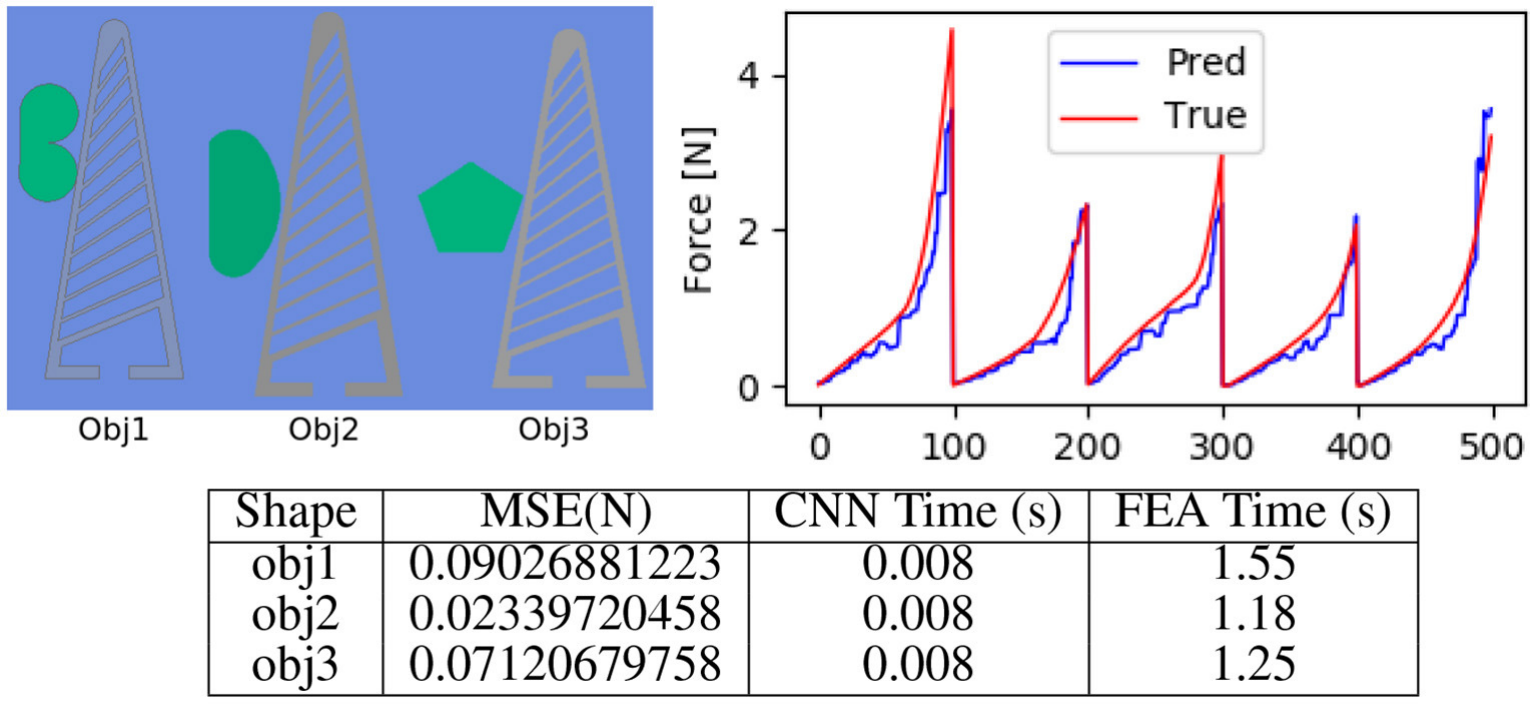
Evaluating shape generalisation. **(Top left)** The three objects with different shape profiles, used for evaluating the approach. **(Top right)** Force (*F*_*x*_) predicted by our approach as compared to the true forces computed using FEA. **(Bottom)** We quantitatively evaluate the mean squared error in the force, which is under 0.1 N. It can be seen that the prediction time by our approach is 0.008 s, which makes it suitable for real time control. For FEA we compute the mean time over 100 Frames on a workstation with i5 (6th generation) 8 GB RAM, 256 GB SSD Windows 10 machine running ANSYS 19.3. Note that the systems used for benchmarking FEA (CPU implementation) and CNN (GPU implementation) are different, which might not be a fair comparison however our approach is over 150 times faster and could be used in realtime, the main reason for the boost is ability of deep learning libraries to parallelly process image data.

**Figure 7:**
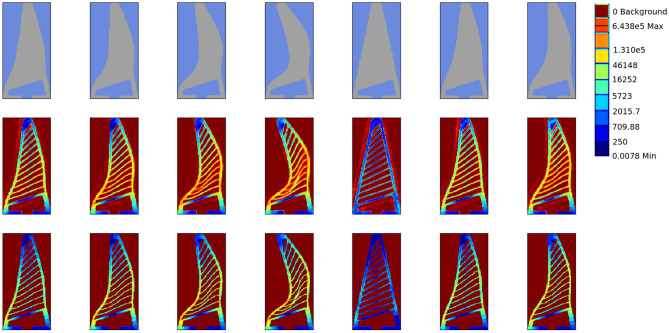
Stress profiling. We qualitatively evaluate the stress profile predicted by our approach in contrast to that generated by FEA simulation. **(Top)** The input object deformation image to the network, **(Middle)** The stress map predicted by our network. **(Bottom)** The ground truth stress map obtained by FEA. The stress values are on logarithmic scale and normalised between [0, 1]. Note that these test shapes are not previously seen by our network in the training data. Our approach is able to reconstruct the fine rib structure from deformation silhouette.

We evaluated our approach on 2,000 number of data samples generated by the FEA setup for three shapes as described in Figure 4 which were different from the two shapes which our network was trained on. In Figure 6, we showcase the results for force predictions. The qualitative stress maps predicted by our network could be visualised from Figure 7, while the quantitative results are summarised in Figure 6. It could be seen that even though the network has not been trained on the three test shapes, it is able to predict the stress and contact forces correctly. This shows the efficacy of the network and the ability to generalise to arbitrarily shaped object, which is crucial for real world applications.

### 5.2. Material Generalisation

**Figure 8:**
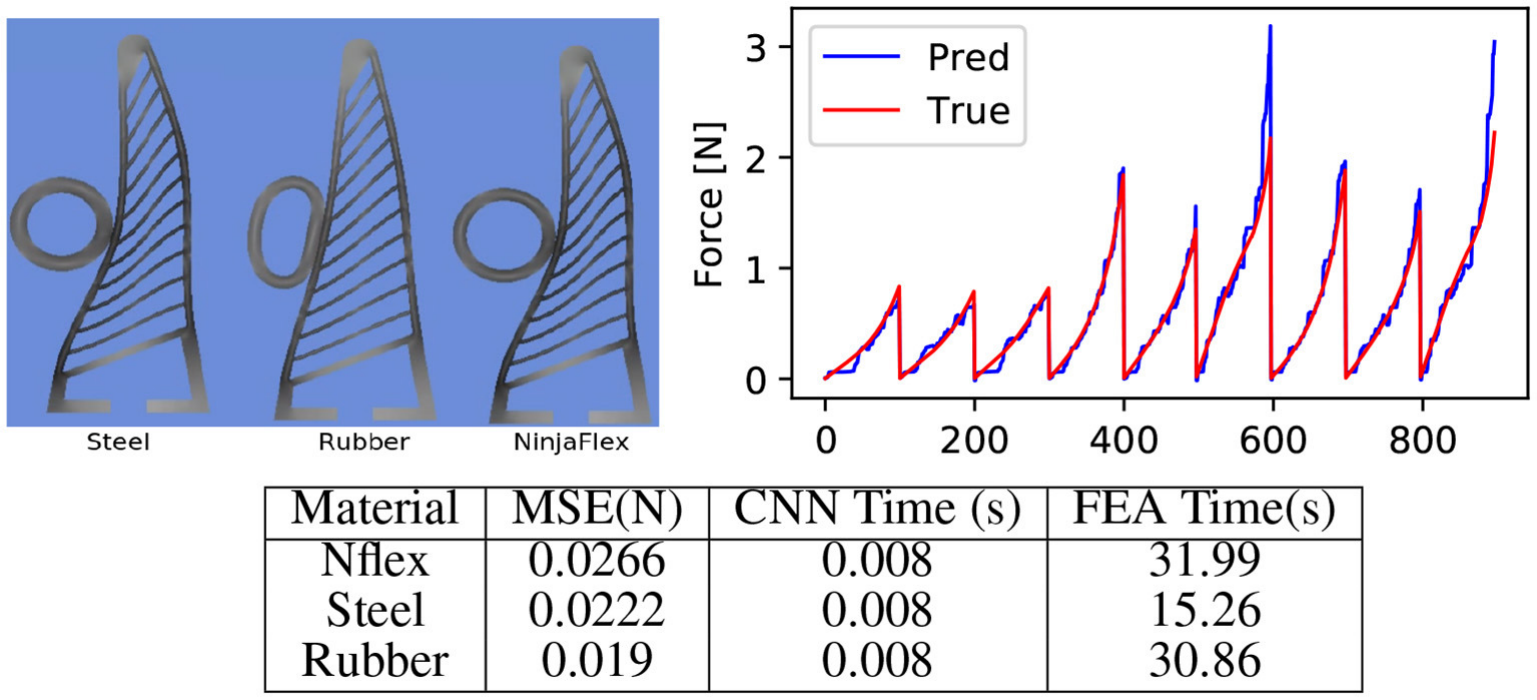
Evaluating material generalisation. **(Top-left)** We additionally evaluate our approach on a ring shaped object made of rubber and steel material having trained only on steel objects (square and cylinder). Note the difference in the stress profile generated by a rubber ring as compared to other material. However, the deformation of the Fin Ray finger is very less in rubber. This confirms our hypothesis that the deformation image indeed captures stress on the gripper. **(Top-right)** Force prediction by our approach as compared to the true fore obtained by the FEA on materials the three materials, which showcases the efficacy of our approach. **(Bottom)** The effect of different material less to the force prediction as the deformation image captures most of the force information. Note that the time taken by FEA to simulate the soft materials significantly large on the other hand, for the learning based approach remains unaffected with the material. This showcases the effectiveness of data driven approaches as surrogate for numerical simulation approaches.

**Figure 9:**
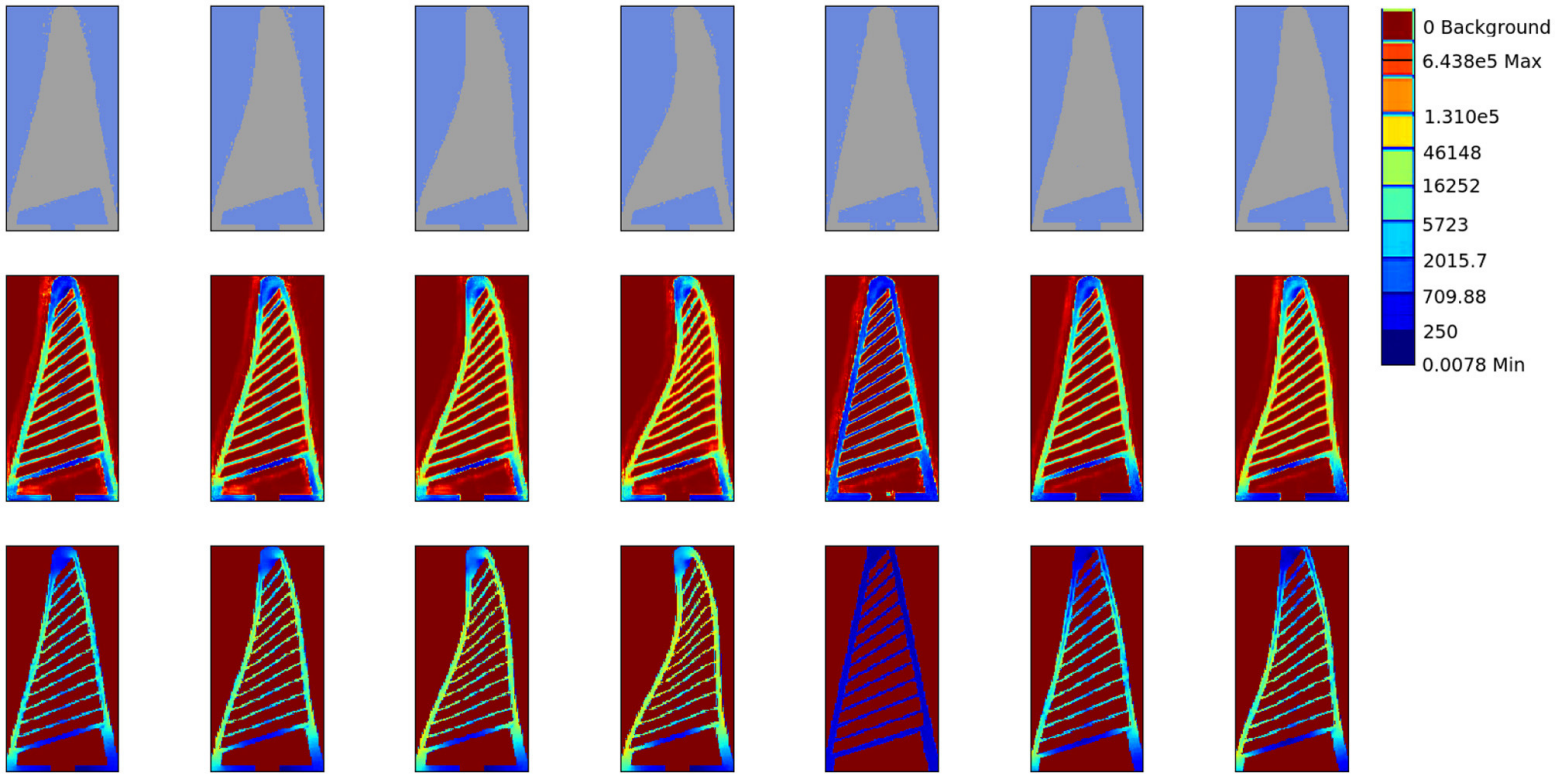
Stress profiling for soft objects. **(Top)** The input object deformation image to the network, **(Middle)** The stress map predicted by our network. **(Bottom)** The ground truth stress map obtained by FEA.

We next evaluate ability of the network to generalise over different materials. We test the network on ring shaped objects made of three different materials namely structural steel, NinjaFlex and neoprene rubber without fine tuning or retraining the network. It can be seen in Figure 8 that our approach easily generalises to different materials. This demonstrates that visual measurements in form of deformation images are sufficient to predict forces and stress maps (Figure 9), with a low mean squared error (Figure 8).

### 5.3. Real World Experiments

**Figure 10:**
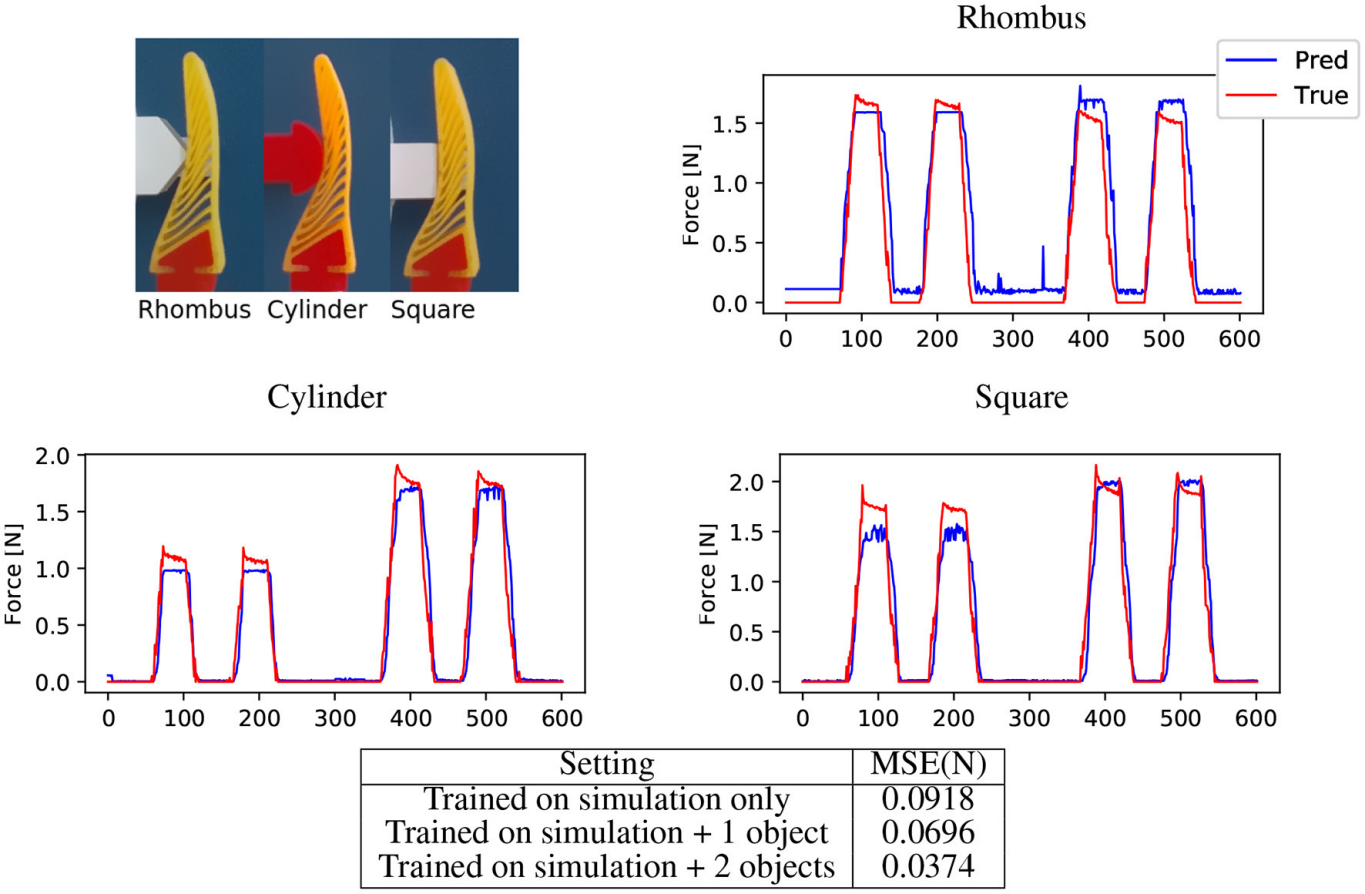
Force prediction for real objects. **(Top-left)** Cropped input images showing three test objects. **(Top-right, Middle-left, Middle-Right)** Force predictions for rhombus, cylinder and square respectively by network trained on simulation data appended with cylinder, rhombus, and square as context object. We compare the predictions with the measurements obtained by the force sensor. Note the incorrect predictions **(Top-right)** in the absence of the force, this is due to the incorrect color segmentation. **(Bottom)** We here present the mean squared error in force on real data. While the network is able to generalise to real data, adding the context object reduces the domain gap and improves the prediction significantly. From the table, we can see that adding data form one object as context results in acceptable performance.

This evaluation compares the network results to real experimental data. A custom test rig is used to monitor the resultant forces acting upon an object under gripper displacement. It is worth noting that this experiment looks at forces in *Z*, with the gripper being displaced vertically. Conversely the FEA experiments were configured primarily around the forces in *X*, with the displacement of the object being locked to that direction. The gripper is the same design as the one used in the aforementioned FEA generated videos. The NinjaFlex gripper is printed directly using a Luzbot Taz 6 (Fargo Additive Manufacturing Equipment 3D, LLC). The objects tested are also produced using 3D-printing, though these are made from rigid ABS. Three objects where used in these tests, with the designs (Figure 10) mimicking some of the aforementioned training objects; namely a 20 mm cylinder and a 15 mm square at 0 and 45°.

**Figure 11:**
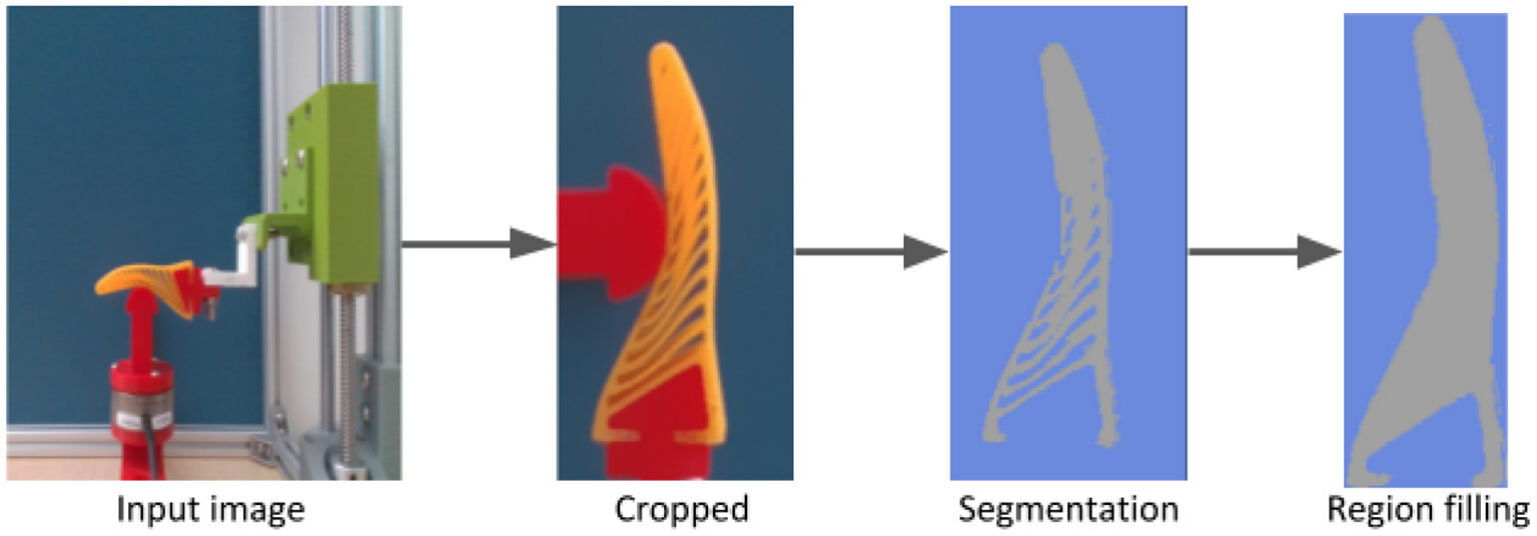
Segmentation Pipeline for real lab experiment. Before feeding the input image to the CNN, we pre-process the image, this image pre-process pipeline consists of cropping followed by a color based segmentation and region filling to fill holes and a final cropping and resizing. This process aims to minimise the domain gap between simulation and real images. However, due to hand-crafted threshold parameters is acceptable to illumination variations. Although we do not focus on bridging this domain gap, we refer the reader to employ recent domain randomisation (Zakharov et al., 2019) and adaptation (James et al., 2019) approaches for optimal results.

**Figure 12:**
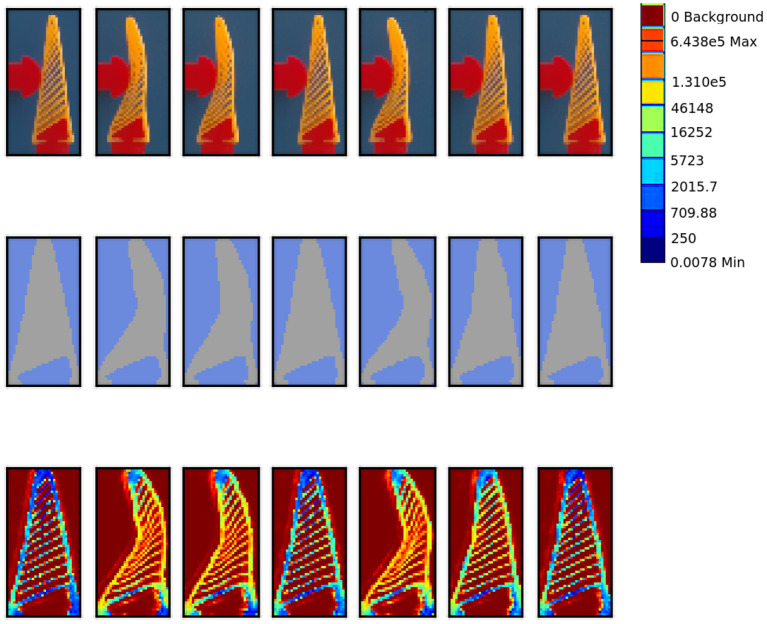
Stress estimation for real objects. We showcase the stress maps predicted by our network for real objects without retraining. **(Top)** Cropped image as input, **(Middle)** Deformation image obtained after the image pre-processing module, this is fed to the network **(Bottom)** The stress map predicted by our network. Note that the true stress map could not be obtained for the real data using FEA thus we only present the predictions by our network.

The test procedure is controlled via a Simulink (The MathWorks, Inc.) model. The model takes the data from the force/torque sensor (Schunk Mini40) and syncs it with RGB video captured on an Intel Realsense D435i camera. This system is synced to 30 frames per second (FPS), with each frame having a corresponding data point. The test procedure consists of lining the object and gripper in one of three positions relative to the base of the gripper (20, 30, 40 mm), similar to the FEA procedure. The vertical axis is driven so that gripper is the positioned around 0.5 mm above the surface of the object. The test procedure is then carried out. The gripper is lowered 10 mm onto the object, held there for 1 s before retracting to its original position. This procedure is repeated once more during the recording, resulting in two cycles per each 10 s video. The test is repeated at the three reference locations with each object. For the sampling rate of 30 FPS used to synchronise images and force measurements, this 10 s video results in 300 images for each object. This raw images are processed with our image processing pipeline Figure 11 to achieve a silhouette of the finger similar to FEA simulations. Although the image processing aims to match the real images with the simulation images, still these images have significant domain gap due to segmentation errors. To minimise this gap we add some random salt-and-pepper noise to the input images while training the network. Furthermore, we append some context images from real objects to the network while training, in Figure 10 it could be seen appending these context images helps is bridging this domain gap. Moreover, approaches from the very recent sim2real literature, for domain randomisation (Zakharov et al., 2019) and domain adaptation (James et al., 2019) could be utilised to further reduce this domain gap in a more principled manner. Figure 10 shows the results of the predicted resultant force with real objects, a estimated stress map is also produced (Figure 12) just as was the case with the purely simulated data.

### 5.4. Evaluating Robustness Toward Partial Occlusions

Since our proposed setup relies on an external camera to observe the gripper deformations for predicting the force and stress values, the performance could be affected by partial occlusions of the gripper from the object being grasped or other objects present in the environment. Thus, in this section, we aim to evaluate the robustness of our approach toward partial occlusion by conducting additional simulation experiments, where a circular object placed randomly selected position is partially occluding the observed image. Given such noisy observations the network is tasked to predict the correct force and stress values. We categorise the severity of the occlusion in four classes namely: none, low (10–30%), medium (40–80%), and high (≥80%), based on the fraction the occluding circle is covering out of the image width. For example in low occlusion, the diameter of the occluding circle is 10–30% the width of the image. The rational behind selecting the percent width as occlusion metric is that the width of the image is half the size of the height and thus contributes more toward the shape of the gripper.

**Figure 13:**
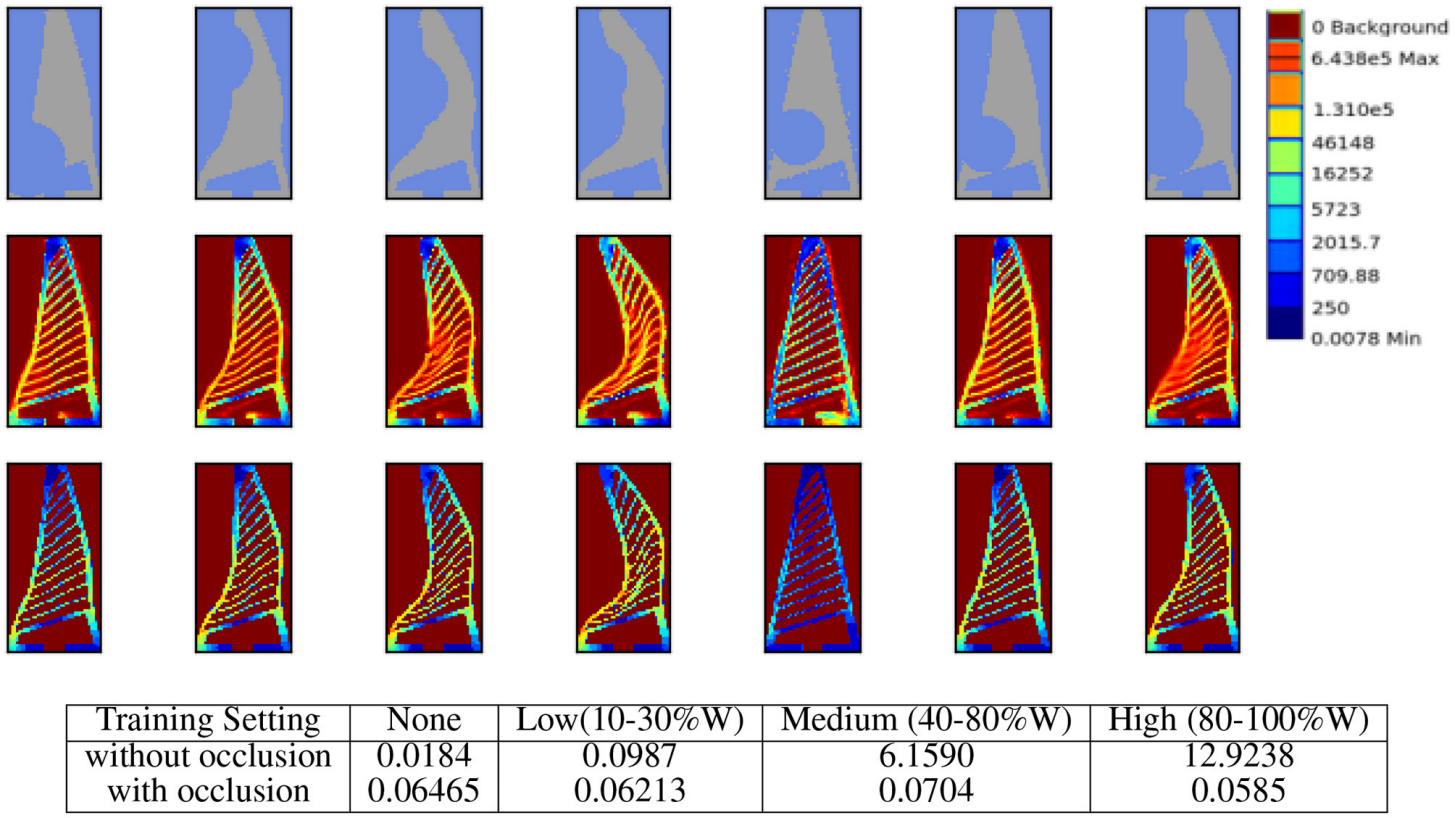
Response to partial occlusion. **(First-row)** Input images with medium to high levels occlusion. **(Second-row)** Stress-map predicted by the approach in spite of occlusion. **(Third-row)** The ground truth stress maps generated from FEA. **(Bottom)** Quantitative results on force predictions under partial occlusions with and without retraining the network. Rows of the table denote the training setting i.e., with or without occlusion. While the columns of the table denote the various level of occlusions in while testing the network. The occlusion is simulated by a circle with diameter as fraction of image width (W) placed at randomly selected coordinates. For the medium level of occlusion, the diameter of the occluding circle is between 40 and 80% width of the image. The results are averaged over 10 trials to maintain consistency since occlusion is selected randomly.

We use the similar training and testing setup as described in experiment 5.1 with occlusion applied to observation images. Here we initially evaluate the network trained without any occlusion on various levels of occlusion, then we re-train the network on training data with random level of occlusion and evaluate again on the test set with different occlusion levels. The results are presented in Figure 13. It can be seen from the table in Figure 13 that even without seeing any occlusion in the training set, the network is able to tackle low levels of occlusions, this is due to the fact that the network is trained on just the silhouette of the gripper and does not directly observe the fine ribs in the input images. Finally, the retrained network is able to cater for even severe levels of occlusion. The stress map prediction results are presented in the top of Figure 13.

### 5.5. Generalisation to Viewpoint Variation

**Figure 14:**
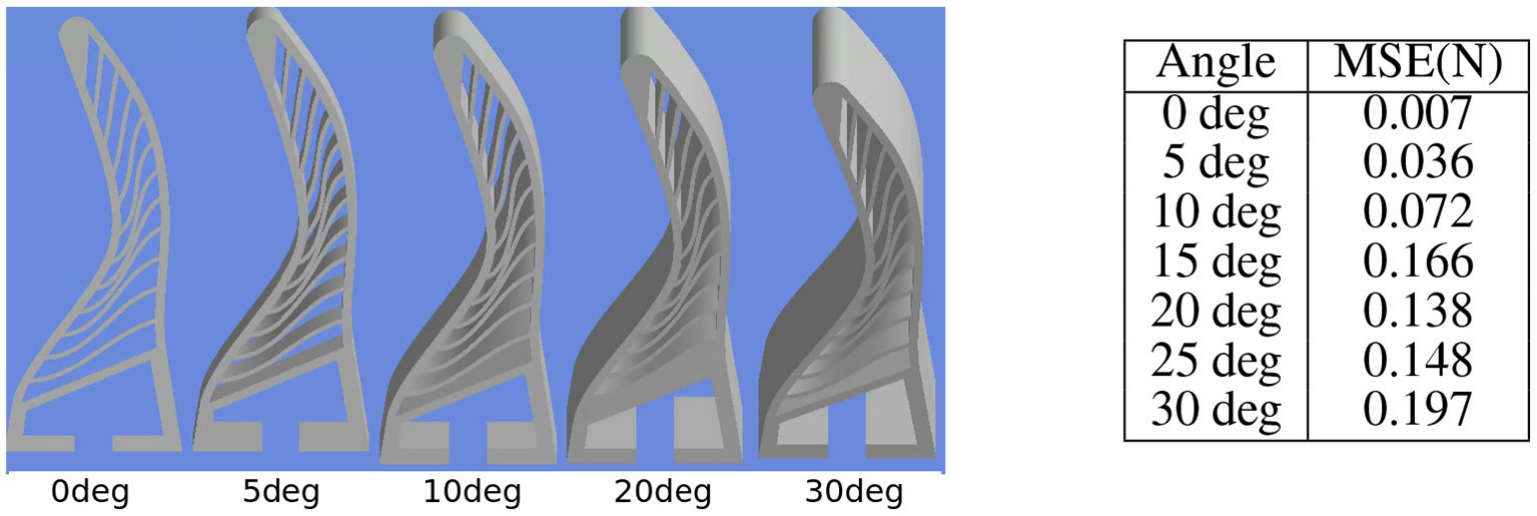
Effect of viewpoint variation on the approach. **(Left)** Input deformation images from FEA by varying camera pitch. **(Right)**. Mean squared error in force predictions encountered by the approach on the test set without any retraining. It can be seen that the approach is able to tackle pitch difference of 10° without retraining.

We further aim to evaluate the viewpoint generalisation of the approach. Here, we collect a test dataset of test object 1 from experiment 5.1, by varying the pitch of the camera from 0 degree (front facing) to 30° in the step of 5° as shown in Figure 14 (left). We compute the force predictions by our network on this test set without any retraining and compute the mean squared error with the ground truth force given by the FEA. It can be seen from the Figure 14 (right) that out approach shows is able to tackle the angle variations below 10°, however for larger camera variations the error is high. However, the approach could easily be retrained for the desired camera configuration.

## 6. Discussions and Conclusion

The primary evaluation for the performance of the proposed system focused on the generalisation of the system with simulated data with regards to variations in object geometry and material. The procedure highlighted in section 5.1 set out to demonstrate the shape generalisation aspect of the system. From the plotted data it is observed that predicted data fits the general trend of the force spikes closely, with a MSE ranging from 0.02 to 0.09 (Figure 6). This is despite the object geometry being new to the system. This close fit demonstrates that the system is indeed capable of variance in shape when using FEA test videos generated in the same way as the training videos.

Part of the desire to use FEA generated data over a purely experimental data was due to the ability of the ANSYS package to produce a colour coded stress map of the gripper. Our system in using this training data is able to produce a stress profile for the gripper under deformation with an unknown object. It can be seen from the stress prediction Figures 7, 9 that our approach is able to truly capture the stress distribution in the gripper. This is crucial in designing grippers with distributes stress profiles that can grasp delicate objects such as fruits without bruising them.

Another reason for the use of FEA training data is due to the ease at which this data can be generated. Around 20,000 data samples were used in the training, with a further 4,000 for the six test samples. Despite the time taken to generate the data from simulation, the process remains faster than generating this experimentally and offers more flexibility in varying object shape and material. The FEA set-up could also be reconfigured for other designs or a change in gripper material. This approach did however encounter some issues, namely excessive local stress causing the simulation to fail. This can be countered by increasing the mesh density or rounding of edges as was done on the square objects described in section 3.1.2. Further refinement of the FEA model, particularly the NinjaFlex material model, may eliminate this issue, but was not the focus of this work.

The other aspect of the simulated data tests examined the systems ability to generalise across different materials. The training data used rigid objects, with the material defined as structural steel. The system therefore has not been trained with the specific intention of adapting to material variance. Despite this, the system has shown in section 5.2 that it is able to generalise for the different materials examined. In the case of the structural steel sample there is effectively no deformation of the object and it performs in the same way as the training data, with a MSE of 0.02. With the NinjaFlex sample there is some object deformation, though as the walls and ribs of the gripper are thinner than the hollow ring, this deformation is quite small. The results therefore largely follow that of structural steel and the training data (MSE 0.03), which has already been shown to be accurate. The final sample is defined as neoprene-rubber and therefore presents significant object deformation. Even in this example, the resultant force profile is predicted accurately, with a MSE of 0.02. This generalisation is due to the fact that the deformation of the ribs is the focus of the system, which remains consistent in cases such as the neoprene rubber where much of the contact force is dissipated into the deforming object rather that the fin ray structure. The result is a lower, but equally well predicted, resultant force from the soft object. Qualitatively the predicted stress profile by the network accurately captured the true stress distribution computed by the FEA process even for the unseen materials.

The previous experiments took place using simulated training and test data. Whilst the results are positive, this does not automatically equate to the system being able to perform under real world conditions. The procedure detailed in section 5.3 demonstrates that the system can indeed perform well on real world data, despite being predominately trained with simulated results. The MSE when the system is purely trained on simulated data is determined as 0.092. When a single real data sample is added into the training, this MSE is reduced to 0.07; with an additional reduction to 0.037 when two lab samples are used. With this comparatively small about of real world data capture, the system is able to accurately predict the resultant forces. The system could naturally have been train on purely experimental data, though as referenced this would have been highly time consuming. Furthermore, predicting stress map would not have been possible in the absence of dense sensory data. Prediction of stress maps could be vital for grasping of delicate objects, allowing the grasp to be examined for minimising excessive local force accumulation and improve the FRE design.

The primary reason for the machine learning approach of this system was to increase the speed at which resultant force and stress mapping can be carried out on a Fin Ray finger; with the aim of future inclusion in a system with real-time vision based grasping force feedback. Whilst the data generation and training stages were time consuming, the trained CNN takes just 8 ms to process a frame.Which makes it suitable for real-time control applications, though the application of this is outside of the scope of this work. As discussed in section 2, there are numerous issues with sensor based approaches. The non-linear behaviour, a result of the gripper's layer jamming fully compliant structure, make analytical techniques difficult to implement. With a simpler geometry, analytical techniques alongside nodal sensors may be feasible, though this would impact the behaviour of the gripper, likely reducing its effectiveness. The speed at which the trained CNN operates, provides an alternative vision based solution which does not impact the behaviour of the gripper.

This work presented a novel approach to determine the contact force of a soft-gripper. The approach presented successfully utilised simulated data to train a CNN. The system has been verified on using both simulation and experimental data, with both showcasing promising results. The system makes use of the exposed geometry of a layer jamming FRE gripper, resulting in a sensor-less system which does not impact the performance of the soft finger, as would be the case where sensors or other means are used. The simulation also allows for the generation of estimated contact stress maps. The system presented could be incorporated into control system which can in real time determine the contact force of the gripper based on it's visible deformation.

## Data Availability Statement

The raw data supporting the conclusions of this article will be made available by the authors, without undue reservation.

## Author Contributions

DD was primarily responsible for the generation of the simulated training and testing data. MP and HP were primarily responsible for the training and verification of the CNN. MH served an editorial role in the writing of the paper. KE supervised research and acted in an editorial role during writing. All authors contributed to the article and approved the submitted version.

## Conflict of Interest

The authors declare that the research was conducted in the absence of any commercial or financial relationships that could be construed as a potential conflict of interest.

## Footnotes

^1^ANSYS engineering data sources: general materials, structural steel.

^2^https://ninjatek.com/wp-content/uploads/2019/10/NinjaFlex-TDS.pdf.

^3^ANSYS engineering data sources: hyperelastic materials, neoprene rubber.
